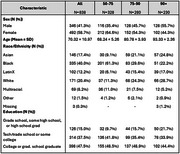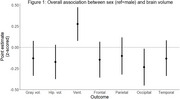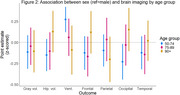# The relationship between sex and brain volume in a diverse sample of older adults differs by age group

**DOI:** 10.1002/alz70856_100440

**Published:** 2025-12-25

**Authors:** Molly R. LaPoint, Rachel A. Whitmer, Sirena Gutierrez, Batool M. Rizvi, María M. M. Corrada, Paola Gilsanz

**Affiliations:** ^1^ Kaiser Permanente Northern California Division of Research, Pleasanton, CA, USA; ^2^ University of California Davis, Davis, CA, USA; ^3^ University of California San Francisco, San Francisco, CA, USA; ^4^ University of California, Davis, Davis, CA, USA; ^5^ University of California, Irvine, Irvine, CA, USA

## Abstract

**Background:**

It is unclear if sex differences in aging‐ and dementia‐related neurodegeneration present in the same manner across the age spectrum.

**Method:**

Analyses utilized data from three harmonized cohorts of racially and ethnically diverse older adults who completed a 3T MRI (*N* = 838; 346 male, 492 female). Measures of total cortical gray matter, hippocampus, ventricular, frontal, occipital, parietal, and temporal lobe volumes were residualized on total intracranial volume separately among male and female participants, and z‐scored. Linear regression models with robust standard errors estimated associations between sex and imaging markers adjusting for age and education level. Possible differences in the effect of sex across age groups were examined using sex by age group interaction terms and age stratified models (age groups: 50‐74, 75‐89, and 90+).

**Result:**

The mean age at scan was 70.3 years (range: 53‐103; Table 1). Overall, female sex was associated with smaller occipital volume (β=‐1.26, 95% CI: ‐2.47, ‐0.05); and larger ventricular volume (β=4.89, 95% CI: 0. 1.33, 8.44) Figure 1). Estimates for the association of female sex with other regions were generally negative but not significant.

Among participants aged 50‐74, female sex was associated with smaller occipital volume (β=‐1.21; 95% CI: ‐2.37, ‐0.05), greater ventricular volume (β=4.75; 95% CI: 2.08, 7.41) and was negatively associated with other brain region volumes though effect estimates were not significant (Figure 1). Sex was not associated with brain region volumes among older age groups. However, the effect estimates of female sex among participants age 90+ meaningly differed and were in the opposite direction than for their counterparts ages 50‐74 when examining occipital volume (β=0.56, 95% CI: ‐0.81, 1.92) and ventricular volume (β=‐1.63, 95% CI: ‐6.80, 3.23; Figure 2).

**Conclusion:**

The relationship between sex and brain region volume may vary by age group. Female sex was associated with smaller occipital and greater ventricular volumes at younger ages, but not at older ages. Further research is needed to elucidate drivers of these sex by age group differences and if they are associated with cognition.